# Economic burden of chronic migraine in OECD countries: a systematic review

**DOI:** 10.1186/s13561-023-00459-2

**Published:** 2023-09-01

**Authors:** Alyaa Eltrafi, Sunil Shrestha, Ali Ahmed, Hema Mistry, Vibhu Paudyal, Saval Khanal

**Affiliations:** 1 School of Pharmacy, College of Medical and Dental Sciences, University of Birmingham, Birmingham, UK; 2 School of Pharmacy, Monash University Malaysia, Bandar Sunway, 47500 Malaysia; 3 Department of Pharmacy Practice, Riphah Institute of Pharmaceutical Sciences, Riphah International University, Islamabad, Pakistan; 4 Warwick Clinical Trials Unit, Warwick Medical School, University of Warwick, Coventry, UK; 5 Health Economics Consulting, Norwich Medical School, University of East Anglia, Bob Champion Research & Education Building, UEA Research Park Rosalind Franklin Rd, NR4 7UQ, Norwich, UK

**Keywords:** Chronic migraine, Cost of illness, Economic burden, Direct costs, Indirect costs, Intangible costs, Productivity losses, Systematic review, Healthcare policy, Resource allocation

## Abstract

**Background:**

Chronic migraine (CM) is a significant neurological condition affecting a substantial portion of the global population. The economic burden of CM includes both direct healthcare costs and indirect costs resulting from productivity losses and intangible impacts on patients’ quality of life. However, there is limited research that comprehensively evaluates all cost components associated with CM, highlighting the need for a systematic review.

**Methods:**

We conducted a systematic literature search in databases including MEDLINE, Embase, and CINAHL to identify studies estimating the cost of illness of chronic migraines. The search was restricted to English language articles published from inception to October 2021, and only findings from Organisation for Economic Co-operation and Development (OECD) countries were included. Methodology features and key findings were extracted from the studies, and reported costs were converted to GBP for cross-country comparisons.

**Results:**

Thirteen cost-of-illness studies on CM from various OECD countries were included in this review. The studies demonstrated substantial variations in monetary estimates, but consistently highlighted the considerable economic burden of CM. Direct costs, particularly hospitalisation and medication expenses, were identified as the highest contributors. However, indirect costs, such as productivity losses due to absenteeism and presenteeism, were often underexplored in the reviewed studies. Additionally, intangible costs related to emotional and social impacts on patients were largely overlooked.

**Conclusion:**

Chronic migraine imposes a significant economic burden on individuals, healthcare systems, and society. Policymakers and healthcare stakeholders should consider both direct and indirect cost components, as well as intangible costs, in developing targeted strategies for effective CM management and resource allocation. Further research focusing on comprehensive cost assessments and sensitivity analyses is needed to enhance the understanding of CM’s economic implications and inform evidence-based healthcare policy decisions. Addressing these research gaps can alleviate the economic burden of CM and improve patient outcomes.

**Supplementary Information:**

The online version contains supplementary material available at 10.1186/s13561-023-00459-2.

## Introduction

Chronic migraine (CM) is a debilitating neurological condition considered one of the most significant non-transmissible diseases worldwide [1]. It is characterised by recurrent, severe throbbing headaches, typically experienced on one half of the head, and accompanied by symptoms such as nausea, dizziness, lack of appetite, extreme photosensitivity, and sensitivity to noise and smells [1]. Although CM has a genetic basis, various internal and external factors can trigger migraine attacks, making it a complex and challenging condition to manage [2].

The global prevalence of CM is estimated to be between 1.4 and 2.2%, impacting millions of individuals and their families worldwide [1]. It is known to cause substantial personal suffering, affecting the overall quality of life and functional ability of those affected. However, beyond the individual level, CM also has far-reaching socio-economic implications for communities and nations at large.

As CM becomes a growing public health concern, it is imperative to comprehend its full economic burden on society. CM poses a significant burden on patients and the healthcare system alike, contributing to substantial financial implications. The financial burden of CM includes both direct costs related to hospitalisation, medical consultations, and medications, as well as indirect costs arising from productivity losses [3]. Indirect costs encompass factors such as absenteeism from work or reduced productivity while at work (presenteeism). Moreover, CM’s domino effects on patients’ quality of life, family dynamics, and emotional well-being also contribute to these substantial indirect costs [4, 5].

While some systematic reviews have previously explored the economic impact of CM and their management [6–8], there remains a need for an updated and comprehensive review that specifically focuses on OECD countries. Such a review would offer insights into the economic challenges posed by chronic migraine in regions characterised by well-established healthcare systems and high-income economies. By examining both direct and indirect costs through a Cost-of-Illness (COI) study, we can gain a more detailed understanding of the economic burden of CM in these countries.

Cost-of-illness (COI) studies provide valuable insights into the economic impact of specific diseases, examining their effects on patients, communities, and entire nations from various perspectives, including payers, healthcare systems, patients, and society at large [9, 10]. In the context of chronic migraine, the indirect costs can be particularly significant, given the condition’s chronic nature and its potential to affect individuals’ ability to maintain their regular daily activities and employment [4, 11, 12]. Consequently, accurately quantifying both direct and indirect costs through COI studies is essential to understanding the full economic burden of chronic migraine on society [9, 10].

Accurate estimates of the economic burden of chronic migraine are of utmost importance as they can significantly inform healthcare policy and decision-making [13]. Stakeholders, including policymakers, patients, and researchers, stand to benefit from a comprehensive understanding of the current burden of chronic migraine and the implications it holds for healthcare policy. Moreover, such insights can underscore the potential benefits of investing in preventive measures and more effective management strategies to alleviate the economic burden associated with chronic migraine.

The Organisation for Economic Co-operation and Development (OECD) is an intergovernmental organisation comprising 38 member countries, aiming to promote economic development and global trade. In this review, we will focus on articles from OECD countries. This selection allows for meaningful and effective comparisons between countries with similar economic backgrounds, enhancing the generalisability of our findings and providing insights into the economic challenges posed by chronic migraine in regions characterised by well-established healthcare systems and high-income economies.

In light of these considerations, our study aims to provide an updated and comprehensive analysis of the cost of illness associated with chronic migraine in OECD countries. By examining both direct and indirect costs, we seek to contribute valuable insights into the economic challenges posed by chronic migraine and inform healthcare policymakers in developing targeted strategies to effectively address its economic consequences. Through this review, we aim to bridge the knowledge gap and offer valuable guidance for policymakers, healthcare providers, and stakeholders in formulating effective measures to alleviate the economic burden of chronic migraine.

## Methods

### Design

This systematic review summarises the economic burden of chronic migraines in OECD countries and analyses the methodology and findings of chronic migraine cost of illness studies. The systematic review was performed following the principles outlined in the Cochrane Handbook for Systematic Reviews of Intervention. The review protocol was registered in PROSPERO (Registration number: CRD42022296395).

### Search strategy and selection criteria

The systematic search was conducted utilising the bibliographic databases MEDLINE, EMBASE and CINAHL (Appendix 1). The search was restricted to articles published in the English language from inception up to October 2021. In addition, a manual search of the reference lists of all included studies and all relevant systematic reviews, found that all relevant studies were identified in our electronic search.

A search strategy was developed to retrieve articles discussing both CMs and COI. The following search terms were used to obtain relevant articles: “cost of illness” or similar terms such as “health expenditures” or “health care costs” or more general terms such as “cost” or “economic burden”, these terms were cross-referenced with the“chronic migraine” related terms such as “transformed migraine” or “chronic headaches”. The search combined the two subjects using the Boolean operator “AND”.

For the systematic review, the relevant articles retrieved from the search were reviewed in accordance with the predetermined inclusion and exclusion criteria. The inclusion criteria covered original studies that investigated the COI of CMs with a calculated monetary value; they were conducted in an OECD country; and published in the English language. The exclusion criteria included: conference abstracts, case reports, systematic reviews, editorials/letters, studies that do not specify chronic migraine, and studies in non-OECD member countries. Cost-effectiveness studies of drugs or treatments which did not calculate the cost without drug or treatment were also excluded.

### Screening and data extraction

Two independent reviewers (AE and SK) screened all titles and abstracts of the articles, and where necessary, the full texts of the articles were screened for the selection criteria. The reference lists of all the selected articles and the relevant systematic reviews were manually screened for any additional inclusions. Any disputes between the two reviewers were settled via a discussion or the input of the third reviewer (VP) when required. Data extraction involved extraction of information such as whether the study was prospective or retrospective and whether they followed incidence or a prevalence approach. A prevalence-based approach measures the cost of illness for a particular time period, typically a year. Whereas, an incidence-based approach involves estimating the lifetime cost of a specific condition. As such, a prevalence-based is considered more accessible and is more common [9]. The information about whether the cost were direct, indirect or intangible were also extracted. The direct costs are the expenses related to the treatment of the condition. Direct costs can be further divided into two categories. Direct healthcare costs include the cost of drugs, hospitalisation, primary care physician visits, emergency room (ER) visits, and diagnostic tests. At the same time, direct non-healthcare costs include the cost of transportation to the hospital or appointments, meal costs and childcare. The indirect costs refer to productivity loss due to absenteeism (missing work) or presenteeism (working while sick). The intangible costs were related to emotional and social costs related to the illness. Intangible costs are seldom calculated as they are exceedingly difficult to quantify [9].

All the estimated costs were converted to GBP (£) in 2021 prices in accordance with the 2021 exchange rate for each currency, with adjustments made for inflation over time based on the Consumer Price index (CPI) [7, 14].

In addition to information about the approach and the cost-involved, the following information were also collected: study details, year of valuation, origin country, perspective and sample size.

### Quality assessment

A quality assessment tool (Appendix 4) adapted for COI studies by Molinier et al. was used for the included articles [15]. This tool incorporated ten items. Each item was rated individually with an answer of yes, partial or no. The final score for each study was a tally of each rating received with a total score. Thus, the highest attainable score was 10. The total score for all the studies was also tallied.

## Results

**Fig. 1:**
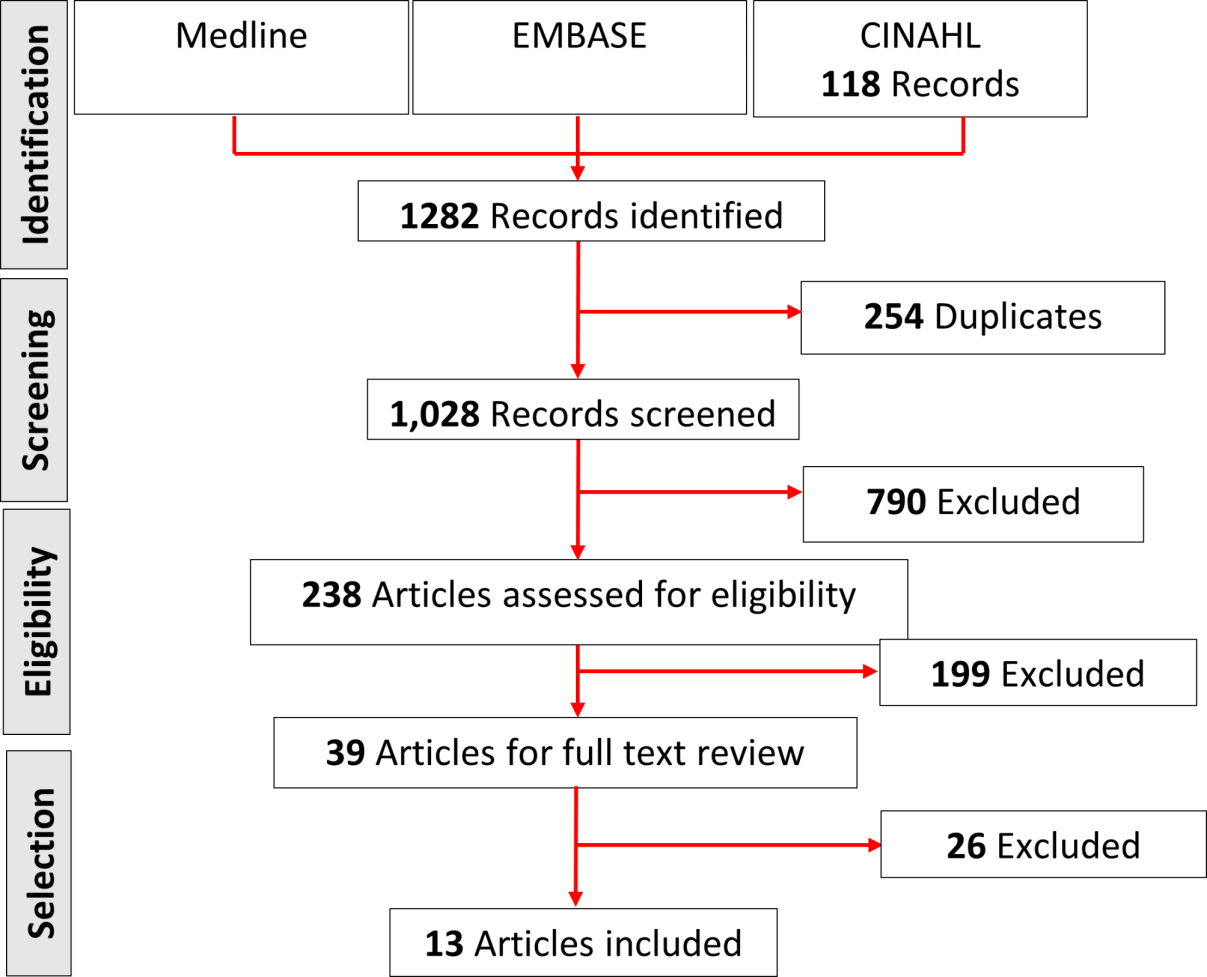
PRISMA flow chart diagram

A total of 1,282 articles were identified from the initial literature search, of which 254 duplicates were identified. After title and abstract screening, 238 articles remained.199 articles were deemed ineligible after applying the inclusion and exclusion criteria. Thus, 39 articles full-text articles were reviewed. However, after applying the inclusion and exclusion criteria to the full text articles, only 13articles were selected for inclusion in this systematic review. The reference list of these thirteen articles and two systematic reviews [7, 16] were screened for additional inclusions which meet the criteria, and no further studies were identified. Figure 1 displays the overall search and selection process for the systematic review.

### Characteristics of the studies

All thirteen COI studies utilised a prevalence-based approach; [12, 14, 17–27].

The methods and key findings of the thirteen studies analysed in the systematic review are summarised in Table 1. From the 13 included articles, COI is estimated for nine of the thirty-eight OECD countries, mainly in Europe and North America. In addition, seven of the thirteen (54%) studies calculated CM related costs in the United States. Of the remaining studies, three measured COI in Italy, two in Spain, one in the United Kingdom, one in Sweden and one in Turkey. Thus, approximately 54% of the investigated data is derived from US estimates [12, 14, 17–27].

**Table 1:** Methodology and findings of chronic migraine cost-of-illness studies

Study No.	Author	Year of Valuation	Country	Study Design	Perspective	CM Sample Size	Direct Cost (Average annual cost per patient)	GBP 2021	Indirect Cost	GBP 2021
**1**	Karli N et al.	2004	Turkey	Cross-sectional	Participant & Societal	219	Chronic Tension Headache= **$104.8** Chronic Daily Headache= **$120.7**	CTH= **£93.12** CDH= **£107.82**	N/A	
**2**	Munakata J et al.	2006	United States	Cohort	Societal	359	**$2,357.97**	**£1,973.77**	Mean Annual cost per patient= **$5,392.03**	**4,511.04**
**3**	Serrano D et al.	2005	United States	Longitudinal	Business	374	N/A		Mean weekly per employee= **$143.28**	**125.52**
**4**	Stokes M et al.	2010	United States & Canada	Cross-Sectional	Health Care System	US = 104 CAD = 55	US= **$4,144** Canada= **$1,883**	US= **3648.21** Canada= **3648.21**	N/A	
**5**	Bloudek LM et al.	2010	United Kingdom, Italy, Spain, France & Germany	Cross-Sectional	Health Care System	UK = 57 ITL = 55 FR = 57 GER = 52 ESP = 56	UK= **€3,718.44** France= **€1,579.00** Germany= **€1,495.20** Italy= **€2,648.12** Spain= **€2,669.80**	UK= **4363.12** France = **1852.76** Germany = **1754.52** Italy= **3106.57** Spain= **3132.00**	N/A	
**6**	Stewart WF et al.	2011	United States	Case Study	Business	185	N/A		Estimated Labour Costs per Week= **$13,384**	**10,847.99**
**7**	Berra E et al.	2013	Italy	Cross-sectional	Health Care System	51	**€2250.00**	**2332.75**	N/A	
**8**	Messali A et al.	2013	United States	Cross- Sectional	Societal	103	**$4,943.00**	**3864.34**	Mean indirect costs= **$3300**	**2579.07**
**9**	Silberstein SD et al.	2013	United States	Cross-sectional	Health Care System	128	**$3,155.00**	**2579.07**	N/A	
**10**	Irimia P et al.	2017	Spain	Cross-sectional	Semi-societal	187	**€8,344.30**	**8219.46**	Mean cost per patient/year= **€8,233.9**	**8110.88**
**11**	Gibbs SN et al.	2018	United States	Cross-sectional	Participant (Out-of-pocket)	319	*Median monthly cost= **$720.50**	**587.09**	N/A	
**12**	Negro A et al.	2019	Italy	Cross- sectional	Health Care System	359	**€2037.00**	**1893.56**	N/A	
**13**	Hansson-Hedblom A et al.	2019	Sweden	Cross-sectional	Semi-societal	454	15–24 Headache days= €**4009** 25–31 Headache days= **€5311**	15–24 Headache days= **£3725.88** 25–31 Headache days= **4936.15**	Annual average 15–24 Headache days= **€31,478** 25–31 Headache days= **€52,521**	15–24 Headache days= **£29254.67** 25–31 Headache days= **£48,810.23**

In addition to CM burden, all thirteen studies calculated COI for episodic migraine (EM) as well. The sample size for the EM population was consistently larger. EM is diagnosed in those experiencing less than 15 headache days per month [11]. CM sample sizes in articles analysed ranged from 51 to 454 [21, 27], whereas the EM sample sizes included up to 7,437 participants [26]. However, the significant disparity in sample sizes is reflective of the increased prevalence of episodic migraines [11]. Nevertheless, all findings exclusively determined a significantly higher economic burden associated with CM compared to EM [12, 14, 17–27]. Table 1 presents the monetary values derived from each article and the GBP 2021 currency conversion.

Eleven of the thirteen articles adhered to the ≥15 headache days per month definition of CM [12, 17–20, 22–27].

### Costs assessed

Table 2 displays the cost components considered by all the articles. Again, all the direct costs are calculated from a single participant perspective, whereas indirect costs were estimated from a societal perspective using a human capital approach.

**Table 2:** Cost components of Chronic migraine cost-of-illness studies

Study No.	Author (Publication Year)	Hospital visits	Primary care physician	Specialist visits	Drugs	Procedures	Diagnostic Testing	Laboratory Tests	ER visits	Transport	Childcare	Indirect Cost
1	Karli N et al. (2006)	✓	✓	✓	✓		✓					
2	Munakata J et al. (2009)	✓	✓	✓	✓				✓			✓
3	Serrano D et al. (2013)											✓
4	Stokes M et al. (2011)	✓	✓	✓	✓	✓	✓	✓	✓			
5	Bloudek LM et al. (2012)	✓	✓	✓	✓	✓	✓	✓	✓			
6	Stewart WF et al. (2011)											✓
7	Berra E et al. (2015) ^)^	✓	✓		✓		✓					
8	Messali A et al. (2016)	✓	✓	✓	✓	✓	✓	✓				✓
9	Silberstein SD et al. (2018)	✓	✓						✓			
10	Irimia P et al. (2020)	✓	✓	✓	✓	✓	✓		✓			✓
11	Gibbs SN et al. (2020)		✓		✓	✓			✓	✓	✓	
12	Negro A et al. (2019)	✓		✓	✓		✓					
13	Hansson-Hedblom A et al. (2020)	✓	✓	✓	✓				✓			✓

Four of the thirteen studies estimated the total cost, the sum of direct and indirect costs. The total annual costs ranged from £6,443 to £53,446 (GBP 2021) in patients with CM [17, 27]. Hansson-Hedblom A et al. and Munakata J et al. estimated total annual cost and found indirect costs to be significantly more burdensome than direct cost [17, 27]. Whereas Irimia P et al. obtained a close approximation between the annual direct and indirect costs, with direct costs being £109 higher [25]. Similarly, Messali A et al. also measured higher direct costs but found a more significant difference between the two expense groups [23]. The discrepancy in cost estimates can be attributed to variable methodology and/or the variation in unit costs.

Indirect costs were measured by six of the included articles [17, 18, 21, 23, 25, 27].

Moreover, none of the articles quantified intangible costs. The annual average for indirect costs ranged from £2,579 to £48,810 (GBP 2021) [23, 27]. Hansson-Hedblom A et al. cost was considered to be an underestimation by the researchers as the survey did not include question about the absenteeism in the work [27]. In contrast, Messali et al. reported the lowest annual indirect cost; the findings of this article are considered contradictory due to the low indirect cost estimation [23]. In addition, the sampling method used by Messali et al. is regarded as a contributor to the contrast in findings [23]. Overall, the indirect costs of CM were inadequately investigated.

Direct costs were estimated by eleven of the thirteen articles reviewed [12, 14, 17, 19, 20, 22–27]. The annual average direct costs per patient ranged from £1754.52 to £8,219.46 [20, 25]. The components incorporated to calculate direct healthcare cost differed (Table 2), affecting the total cost estimation. Bloudek LM et al., which estimated the lowest COI in Germany, used public sources for the healthcare unit costs; therefore, the results are susceptible to discrepancies [20].

Most of the studies used a cross-sectional retrospective survey design and included comparison values for episodic migraines. Additionally, studies collected data for three months and extrapolated results to calculate annual costs.

## Discussion

### Summary of the findings

This systematic review presents an in-depth analysis of thirteen cost-of-illness (COI) studies focused on chronic migraine (CM) from various countries. The findings reveal substantial variations in the monetary values estimated, while consistently highlighting the considerable economic burden of CM in all the included studies. Direct costs of CM primarily comprise hospitalisation and medication expenses. Some studies reported chronic tension headache and chronic daily headache as significant contributors to direct costs. However, the investigation of indirect costs remains limited, and intangible costs are often not considered. The quality assessment of the studies indicates that while most studies achieved a reasonable level of quality, there are certain limitations, such as small sample sizes and the lack of disaggregated cost data and sensitivity analyses to test major assumptions.

### Relevance to published studies

The findings of this systematic review are highly relevant and contribute to the existing body of knowledge on the economic burden of chronic migraine (CM). They align with previously published studies, which have also highlighted the substantial economic impact of CM on individuals, healthcare systems, and society as a whole [1, 2]. By synthesising the results of multiple COI studies, this review provides a comprehensive and updated overview of the financial implications of CM.

The identification of direct and indirect costs in the reviewed studies is consistent with other research on CM. Hospitalisation and medication expenses have been consistently reported as major contributors to the direct costs of CM [7, 14, 16, 17]. These findings reinforce the importance of effective management strategies to reduce hospitalisations and reliance on medications, which can help alleviate the economic burden on both patients and healthcare systems.

Moreover, the limited investigation of indirect costs in many of the reviewed studies mirrors the gaps identified in earlier research [3, 28]. By shedding light on this underexplored aspect of CM’s economic burden, this review underscores the need for further attention to productivity losses due to absenteeism, presenteeism, and the impact on daily activities and work responsibilities. Ignoring these substantial indirect costs can lead to an incomplete understanding of CM’s overall economic impact.

Interestingly, some studies in this review reported that indirect costs could surpass direct costs, indicating that the economic burden of CM might be even greater than previously assumed [9, 10, 13]. This observation emphasizes the significance of accounting for all cost components in COI studies and provides an impetus for researchers and policymakers to address both direct and indirect cost factors when evaluating the economic burden of CM.

By emphasizing the importance of considering intangible costs associated with emotional and social hardships for patients with CM, this review echoes the concern expressed in previous research [13]. Chronic migraine can significantly impact the quality of life, mental well-being, and social functioning of affected individuals. Future studies should incorporate measures to assess and quantify these intangible costs, as they have far-reaching implications for patients and society.

### Strength and limitation of the studies

The reviewed studies demonstrate several strengths, including clear definitions of CM, careful description of epidemiological sources, and appropriate valuation of unit costs. The majority of studies also provided well-explained methods and presented their results consistently with their methodologies. The use of cross-sectional, cohort, and longitudinal designs further enhances the understanding of CM’s economic burden over time.

However, the limitations of the studies should be acknowledged. The small sample sizes in most studies may impact the generalisability of the findings. Future research with larger sample sizes would provide more robust and representative estimates of the economic burden of CM. Additionally, the predominant focus on direct healthcare-related costs overlooks the significant impact of indirect costs, which can be substantial in CM. Future studies should aim to include a comprehensive assessment of both direct and indirect costs to provide a more holistic understanding of the economic burden associated with CM. Furthermore, the lack of sensitivity analyses to test major assumptions may introduce uncertainties in the cost estimations. Incorporating sensitivity analyses in future studies would improve the credibility of the findings.

### Further implications and research

The economic burden of CM has far-reaching implications for patients, healthcare systems, and society. Policymakers and healthcare stakeholders can use the insights from this systematic review to develop targeted strategies for effective migraine management, ultimately reducing the economic burden on individuals and society as a whole. Understanding the direct and indirect costs associated with CM can inform decision-making on resource allocation, funding, and reimbursement for CM treatments and interventions.

To enhance the understanding of CM’s economic burden, future research should focus on comprehensive assessments that include both direct and indirect costs, including intangible costs related to emotional and social hardships for patients. Sensitivity analyses should also be incorporated to account for uncertainties in cost estimations. Additionally, further investigations into the impact of CM on productivity losses and the ability of prophylactic treatments to prevent migraine attacks can contribute to more effective management strategies and improved patient outcomes.

## Conclusion

In conclusion, this systematic review provides valuable insights into the economic burden of chronic migraine, highlighting the significant direct and indirect costs associated with the condition. Policymakers, healthcare stakeholders, and researchers can use these findings to make informed decisions about migraine management strategies and resource allocation. Addressing the limitations identified in the reviewed studies will strengthen the quality and reliability of cost-of-illness studies on chronic migraine and contribute to a more comprehensive understanding of the economic impact of this debilitating condition. By considering all cost components and conducting sensitivity analyses, future research can further inform decision-making and improve the management and support systems for CM patients.

### Electronic supplementary material

Below is the link to the electronic supplementary material.

Supplementary Material 1

## Data Availability

The datasets during and/or analysed during the current study are available from the corresponding author on reasonable request.

## Abbreviations

COI: Cost-of-illness
CM: Chronic Migraine
EM: Episodic Migraine
OECD: Organisation for Economic Co-operation and Development
ER: Emergency Room
