# Correction: Economic burden of chronic migraine in OECD countries: a systematic review

**DOI:** 10.1186/s13561-023-00461-8

**Published:** 2023-10-25

**Authors:** Alyaa Eltrafi, Sunil Shrestha, Ali Ahmed, Hema Mistry, Vibhu Paudyal, Saval Khanal

**Affiliations:** 1 School of Pharmacy, College of Medical and Dental Sciences, University of Birmingham, Birmingham, UK; 2 School of Pharmacy, Monash University Malaysia, Bandar Sunway, 47500 Malaysia; 3 Department of Pharmacy Practice, Riphah Institute of Pharmaceutical Sciences, Riphah International University, Islamabad, Pakistan; 4 Warwick Clinical Trials Unit, Warwick Medical School, University of Warwick, Coventry, UK; 5 Health Economics Consulting, Norwich Medical School, University of East Anglia, Bob Champion Research & Education Building, UEA Research Park Rosalind Franklin Rd, Norwich, NR4 7UQ UK

**Correction:** ***Health Econ Rev*** **13, 43 (2023)**

10.1186/s13561-023-00459-2

Following publication of the original article [1], the authors corrected the errors in the affiliations of Ali Ahmed, Hema Mistry, and Saval Khanal.

Correct affiliations are as follows:

**Ali Ahmed**

• *School of Pharmacy, Monash University Malaysia, Bandar Sunway, 47500, Malaysia*.

• *Department of Pharmacy Practice, Riphah Institute of Pharmaceutical Sciences, Riphah International University, Islamabad, Pakistan*.

**Hema Mistry**

• *Warwick Clinical Trials Unit, Warwick Medical School, University of Warwick, Coventry, UK*.

**Saval Khanal**

• *Health Economics Consulting, Norwich Medical School, University of East Anglia, Bob Champion Research & Education Building, UEA Research Park Rosalind Franklin Rd, NR4 7UQ, Norwich, UK*.

The affiliations have been updated above and the original article [1] has been corrected.
