# Supplementary material for: Economic burden of chronic migraine in OECD countries: a systematic review
Source: Health Econ Rev. 2023 Sep 1;13:43. doi: 10.1186/s13561-023-00459-2 (PMC10472624; doi:10.1186/s13561-023-00459-2)
Supplement: Supplementary file 1 — Supplementary Material 1 [file 13561_2023_459_MOESM1_ESM.docx]

**Appendices**

**Appendix 1a. MEDLINE Search Strategy**

| Database: Ovid MEDLINE(R) <1946 to October Week 3 2021> |
| --- |
| Search Strategy: |
| 1 Chronic migraine.mp. (2077) |
| 2 Transformed migraine.mp. (186) |
| 3 Frequent migraine.mp. (100) |
| 4 Frequent headache.mp. (173) |
| 5 Chronic Headache.mp. (1398) |
| 6 Chronic daily headache.mp. (771) |
| 7 Chronic tension-type headache.mp. (648) |
| 8 Medication-overuse headache.mp. (777) |
| 9 Drug induced headache.mp. (60) |
| 10 1 or 2 or 3 or 4 or 5 or 6 or 7 or 8 or 9 (5035) |
| 11 Costs.mp. or "Costs and Cost Analysis"/ (264889) |
| 12 Health Expenditures.mp. or Health Expenditures/ (22830) |
| 13 Health Care Costs.mp. or Health Care Costs/ (50923) |
| 14 Cost of illness.mp. or "Cost of Illness"/ (30375) |
| 15 Economic Burden.mp. (10243) |
| 16 Economics*.mp. (490453) |
| 17 Expenditures*.mp. (34297) |
| 18 11 or 12 or 13 or 14 or 15 or 16 or 17 (638171) |
| 19 10 and 18 (195) |
| 20 limit 19 to english language (178) |

**Appendix 1b. EMBASE Search Strategy**

| Database: Embase <1974 to 2021 October 22> |
| --- |
| Search Strategy: |
| 1 Chronic Migraine.mp. (5980) |
| 2 transformed migraine.mp. or transformed migraine/ (6413) |
| 3 Frequent migraine.mp. (203) |
| 4 Frequent headache.mp. (305) |
| 5 Chronic headache.mp. (2800) |
| 6 Chronic daily headache.mp. or chronic daily headache/ (2013) |
| 7 Chronic tension-type headache.mp. or chronic tension headache/ (1506) |
| 8 medication-overuse headache.mp. (1770) |
| 9 drug induced headache.mp. or drug induced headache/ (19246) |
| 10 1 or 2 or 3 or 4 or 5 or 6 or 7 or 8 or 9 (30199) |
| 11 "cost"/ or Costs.mp. (380024) |
| 12 Cost analysis.mp. or "cost benefit analysis"/ (94027) |
| 13 Health Expenditures.mp. (2820) |
| 14 health care costs.mp. or "health care cost"/ (209998) |
| 15 Cost of illness.mp. or "cost of illness"/ (21724) |
| 16 economic burden.mp. (21592) |
| 17 health economics/ or economics*.mp. (298005) |
| 18 expenditures*.mp. (23132) |
| 19 11 or 12 or 13 or 14 or 15 or 16 or 17 or 18 (796213) |
| 20 10 and 19 (969) |
| 21 limit 20 to english language (942) |
| 22 limit 21 to (exclude medline journals and conference abstracts) (3) |

**Appendix 1c. CINAHL Search Strategy**

| **#** | **Query** | **Limiters/Expanders** | Results |
| --- | --- | --- | --- |
| S20 | S10 AND S18 | Expanders - Apply equivalent subjects  Narrow by Language: - English  Search modes - Boolean/Phrase | 116 |
| S19 | S10 AND S18 | Expanders - Apply equivalent subjects  Search modes - Boolean/Phrase | 118 |
| S18 | S11 OR S12 OR S13 OR S14 OR S15 OR S16 OR S17 | Expanders - Apply equivalent subjects  Search modes - Boolean/Phrase | 231,495 |
| S17 | "Expenditures*" OR (MH "Economics") | Expanders - Apply equivalent subjects  Search modes - Boolean/Phrase | 21,889 |
| S16 | "Health economics" | Expanders - Apply equivalent subjects  Search modes - Boolean/Phrase | 7,373 |
| S15 | (MH "Economic Aspects of Illness") OR "cost of illness" | Expanders - Apply equivalent subjects  Search modes - Boolean/Phrase | 10,922 |
| S14 | (MH "Health Care Costs") | Expanders - Apply equivalent subjects  Search modes - Boolean/Phrase | 59,340 |
| S13 | "Health expenditure" | Expanders - Apply equivalent subjects  Search modes - Boolean/Phrase | 1,110 |
| S12 | (MH "Costs and Cost Analysis") OR (MH "Cost Benefit Analysis") | Expanders - Apply equivalent subjects  Search modes - Boolean/Phrase | 54,322 |
| S11 | "cost" | Expanders - Apply equivalent subjects  Search modes - Boolean/Phrase | 173,638 |
| S10 | S1 OR S2 OR S3 OR S4 OR S5 OR S6 OR S7 OR S8 OR S9 | Expanders - Apply equivalent subjects  Search modes - Boolean/Phrase | 3,062 |
| S9 | "Drug induced headache" | Limiters - Published Date: -20211031  Expanders - Apply equivalent subjects  Search modes - Boolean/Phrase | 14 |
| S8 | "Medication overuse headache" | Limiters - Published Date: -20211031  Expanders - Apply equivalent subjects  Search modes - Boolean/Phrase | 533 |
| S7 | "`Chronic tension type headache" | Limiters - Published Date: -20211031  Expanders - Apply equivalent subjects  Search modes - Boolean/Phrase | 337 |
| S6 | "Chronic daily headache" | Limiters - Published Date: -20211031  Expanders - Apply equivalent subjects  Search modes - Boolean/Phrase | 418 |
| S5 | "Chronic headache" | Limiters - Published Date: -20211031  Expanders - Apply equivalent subjects  Search modes - Boolean/Phrase | 643 |
| S4 | "Frequent headache" | Limiters - Published Date: -20211031  Expanders - Apply equivalent subjects  Search modes - Boolean/Phrase | 96 |
| S3 | "Frequent migraine" | Limiters - Published Date: -20211031  Expanders - Apply equivalent subjects  Search modes - Boolean/Phrase | 55 |
| S2 | "Transformed migraine" | Limiters - Published Date: -20211031  Expanders - Apply equivalent subjects  Search modes - Boolean/Phrase | 94 |
| S1 | "Chronic migraine" | Limiters - Published Date: -20211031  Expanders - Apply equivalent subjects  Search modes - Boolean/Phrase | 1,524 |

| **Appendix 2. Quality Assessment tool** | | | | | | | |  |
| --- | --- | --- | --- | --- | --- | --- | --- | --- |
| **Questions** | All Studies | Karli N et al. (2006) | Munakata J et al. (2009) | Serrano D et al. (2013) | Stokes M et al. (2011) | Bloudek LM  et al.  (2012) | Stewart WF et al.  (2011) | Berra E et al.  (2015) |
| 1. Was a clear definition of the illness given? |  | P | 1 | 1 | 1 | 1 | P | 0 |
| 2. Were epidemiological sources carefully described? |  | 1 | 1 | 1 | 1 | 1 | 1 | 1 |
| 3. Were direct/indirect costs sufficiently disaggregated? |  | 0 | 1 | 0 | 0 | 0 | 0 | 0 |
| 4. Were activity data sources carefully described? |  | 1 | 1 | P | 1 | 1 | 1 | 1 |
| 5. Were activity data appropriately assessed? |  | 1 | 1 | 1 | 1 | P | 1 | 1 |
| 6. Were the sources of all cost values analytically described? |  | 1 | 1 | 0 | 1 | 1 | 0 | P |
| 7. Were unit costs appropriately valued? |  | P | 1 | P | 1 | 1 | 1 | P |
| 8.Were the methods adopted carefully explained? |  | 1 | 1 | 1 | 1 | 1 | 1 | 1 |
| 9. Were the major assumptions tested in a sensitivity analysis? |  | 0 | 0 | 0 | 1 | 0 | 0 | 0 |
| 10. Was the presentation of study results consistent with the methodology of the study? |  | 1 | 1 | 1 | 1 | 1 | 1 | 1 |
| **Total score by study** |  |  |  |  |  |  |  |  |
| **Yes (1)** |  | **6** | **9** | **5** | **9** | **7** | **6** | **5** |
| **No (0)** |  | **2** | **1** | **3** | **1** | **2** | **3** | **3** |
| **Partially (p)** |  | **2** | **0** | **2** | **0** | **1** | **1** | **2** |

| **Questions** | All Studies | Messali A et al. (2016) | Silberstein SD et al.  (2018) | Irimia P et al. (2020) | Gibbs SN et al. (2020) | Negro A et al. (2019) | Hansson-Hedblom A et al. (2020) |
| --- | --- | --- | --- | --- | --- | --- | --- |
| 1. Was a clear definition of the illness given? |  | 1 | 1 | 1 | P | 1 | 1 |
| 2. Were epidemiological sources carefully described? |  | 1 | 1 | P | 1 | 1 | 1 |
| 3. Were direct/indirect costs sufficiently disaggregated? |  | 1 | 0 | 1 | 0 | 0 | 1 |
| 4. Were activity data sources carefully described? |  | 1 | P | P | 1 | P | P |
| 5. Were activity data appropriately assessed? |  | 1 | 1 | 1 | 0 | 1 | 1 |
| 6. Were the sources of all cost values analytically described? |  | 1 | P | 1 | 1 | 1 | 0 |
| 7. Were unit costs appropriately valued? |  | 1 | 1 | 1 | 1 | P | 1 |
| 8. Were the methods adopted carefully explained? |  | 1 | 1 | 1 | 1 | 1 | 1 |
| 9. Were the major assumptions tested in a sensitivity analysis? |  | 0 | 0 | 0 | 0 | 0 | P |
| 10. Was the presentation of study results consistent with the methodology of the study? |  | 1 | 1 | 1 | 1 | 1 | 1 |
| **Total score by study** |  |  |  |  |  |  |  |
| **Yes (1)** | **83** | **9** | **6** | **7** | **6** | **6** | **7** |
| **No (0)** | **22** | **1** | **2** | **1** | **3** | **2** | **1** |
| **Partially (p)** | **15** | **0** | **2** | **2** | **1** | **2** | **2** |
